# Complete Genome Sequence of *Bartonella ancashensis* Strain 20.00, Isolated from the Blood of a Patient with Verruga Peruana

**DOI:** 10.1128/genomeA.01217-15

**Published:** 2015-11-05

**Authors:** Jun Hang, Kristin E. Mullins, Robert J. Clifford, Fatma Onmus-Leone, Yu Yang, Ju Jiang, Mariana Leguia, Matthew R. Kasper, Ciro Maguiña, Emil P. Lesho, Richard G. Jarman, Allen L. Richards, David Blazes

**Affiliations:** a Walter Reed Army Institute of Research, Silver Spring, Maryland, USA; b Uniformed Services University of the Health Sciences, Bethesda, Maryland, USA; c Naval Medical Research Center, Silver Spring, Maryland, USA; d U.S. Naval Medical Research Unit No. 6, Lima, Peru; e Universidad Peruana Cayetano Heredia, Lima, Peru

## Abstract

Here we present the complete genome sequence of *Bartonella ancashensis* strain 20.00, isolated from the blood of a Peruvian patient with verruga peruana, known as Carrion’s disease. *Bartonella ancashensis* is a Gram-negative bacillus, phylogenetically most similar to *Bartonella bacilliformis*, the causative agent of Oroya fever and verruga peruana.

## GENOME ANNOUNCEMENT

Members of the genus *Bartonella* are Gram-negative bacilli that are found worldwide and are associated with animal and human diseases (1). The most common diseases associated with *Bartonella* species are trench fever, cat scratch disease, and the biphasic illness Carrion’s disease, which are caused by *B. quintana*, *B. henselae*, and *B. bacilliformis*, respectively (2–4). More recently, *B. henselae* and *B. quintana* have been associated with bacillary angiomatosis, and more than eight *Bartonella* species have been associated with either febrile illnesses or endocarditis (2, 4, 5).

In a 2003 study involving 127 patients with verruga peruana in the Peruvian Ancash mountain area, two patients were found to be infected with a *Bartonella* species distinct from any known *Bartonella* species. This determination was based on the sequences of the *gltA*, *rpoB*, *ftsZ*, *groEL*, and *rrs* genes and the 16s-23s intergenic internal transcribed spacer (ITS) region. The genetic differences and culture characteristics of the strain meet the taxonomic criteria to classify this non-*B. bacilliformis* pathogen as a new species, designated *Bartonella ancashensis*. The nomenclature has been accepted by the *International Journal of Systematic and Evolutionary Microbiology*, and *B. ancashensis* strain 20.00 was accepted by both ATCC and DSMZ for archiving and distribution for research use (6–8).

The genome of *B. ancashensis* strain 20.00 was sequenced using Roche 454 next-generation sequencing technology (Roche 454 Life Sciences, Branford, CT). A total of 198,990 filtered reads consisting of 57.4 Mb of data were assembled into 94 contigs with average sequence coverage of 41.7× using GS Assembler software (Newbler) version 2.5.3. Final assembly was verified using a whole-genome AflII restriction map generated with the Argus system (OpGen, Gaithersburg, MD) (9). *B. ancashensis* strain 20.00 has a circular genome of 1,466,048 bp and has a G+C content of 38.4%, which is similar to the genome of the *Bartonella* prototype strain, *B. bacilliformis* KC583 (NC_008783.1), which is 1,445,021 nucleotides in length with a G+C content of 38.2%.

Whole-genome annotation was performed using RAST (Rapid Annotation using Subsystem Technology) (http://www.nmpdr.org/FIG/wiki/view.cgi/FIG/RapidAnnotationServer); structural and functional annotation was completed using the IGS Annotation Engine (http://ae.igs.umaryland.edu/cgi/index.cgi). Genome annotations were reviewed and finalized using Genome Viewer (http://www.nmpdr.org/FIG/wiki/view.cgi/FIG/GenomeViewer) and Manatee (http://manatee.sourceforge.net/) (10, 11). The *B. ancashensis* strain 20.00 genome contains 1,346 putative protein-encoding genes, of which 79.1% are found to have homologs in *B. bacilliformis*. Like *B. bacilliformis*, *B. ancashensis* strain 20.00 encodes flagellar proteins. However, unlike *B. bacilliformis*, *B. ancashensis* strain 20.00 encodes VirB/D4 type IV secretion system proteins most similar to those of *B. clarridgeiae* and *B. rochalimae*, both of which have been implicated in human disease. Further, *B. ancashensis* possesses a family of virulence-modulating proteins which are present only in *B. australis* and human-pathogenic *Leptospira* species (12–16).

Here we have reported the first complete genome sequence for the new pathogen *B. ancashensis*. Further *B. ancashensis* genome studies and comparisons will elucidate factors involved in virulence and pathogenicity of not only *B. ancashensis*, but also the *Bartonella* genus as a whole.

### Nucleotide sequence accession number.

This whole-genome shotgun project has been deposited at DDBJ/EMBL/GenBank under the accession number CP010401.
